# Development of Compartment Syndrome after Radial Artery Puncture in a Patient with Acute Hypoxemic Respiratory Failure due to COVID-19

**DOI:** 10.1155/2022/8241057

**Published:** 2022-04-23

**Authors:** Orlando Garner, Krishidhar Nunna, Andrea Braun

**Affiliations:** Baylor College of Medicine, Houston, TX, USA

## Abstract

A 71-year-old man who was recently hospitalized for COVID-19 pneumonia complicated by acute hypoxemic respiratory failure and severe ARDS requiring noninvasive ventilation was transferred to our hospital from a rehabilitation facility for new onset right wrist and hand pain and swelling which had been attributed to arterial thrombosis and empirically treated with therapeutic anticoagulation. He developed numbness and paralysis in his right hand and was diagnosed with right forearm compartment syndrome requiring emergent fasciotomy. After a prolonged hospital stay complicated by respiratory failure requiring mechanical ventilation, he was discharged with improved, but not resolved, sensorimotor deficits. Arterial blood gas sampling is commonly performed in patients with acute hypoxemic respiratory failure, for assessment of oxygenation and acid-base status. It is considered a benign procedure, but it can lead to serious complications, such as bleeding and compartment syndrome. Risks and benefits of any procedure need to be weighed carefully and less is often more. Compartment syndrome is characterized by the 5 P's—pain, pallor, paresthesia, pulselessness, and paralysis.

## 1. Introduction

Iatrogenic complications cause significant morbidity and mortality worldwide. A recent meta-analysis found that approximately 22,165 deaths in hospitalized patients in the United States are preventable each year [1]. In contrast, an Institute of Medicine report from 2000, “To Err is Human,” attributed roughly 98,000 in-hospital deaths per year to human error [2].

The COVID-19 pandemic has substantially increased the number of intensive care unit (ICU) patients with hypoxemic respiratory failure whose oxygenation and acid-base status are monitored by arterial blood gas (ABG) analysis. Though obtaining an ABG is a common procedure in the emergency department and the ICU and generally considered harmless, complications can occur, and physicians need to weigh the risks and benefits of obtaining an ABG, including whether the result will change patient management, rather than routinely performing this test in hypoxemic ICU patients. Potential complications after arterial puncture include bleeding, hematoma, arterial thrombosis, arterial pseudoaneurysm, and arteriovenous fistula development. Compartment syndrome is a devastating complication that has been described in only a few cases after arterial puncture for blood gas analysis or radial artery catheterization. It can lead to permanent nerve and muscle damage with limb paralysis and loss of hand and arm function. Early recognition and treatment can reduce the associated morbidity significantly.

## 2. Case Presentation

A 71-year-old man with a medical history of coronary artery disease requiring PCI (percutaneous coronary intervention) years ago, hypertension, hyperlipidemia, gout, and benign prostatic hyperplasia was transferred to our facility from a long-term acute care facility (LTAC) with a 2-day history of right wrist and hand pain and swelling. 18 days earlier, the patient had been diagnosed COVID-19 viral pneumonia and was admitted to another hospital with acute hypoxemic respiratory failure requiring oxygen administration via high-flow nasal cannula and noninvasive bilevel positive pressure ventilation (BIPAP). After a 16-day ICU stay and treatment with dexamethasone and empiric antibiotics, he was transferred to a LTAC because of persistent high oxygen requirements and the need for prolonged noninvasive ventilation with high-flow nasal cannula and BIPAP. He had undergone frequent ABG sampling from both radial arteries, as well as multiple venous blood draws and peripheral intravenous line placements on both the hands and arms, though he never had an arterial line placed.

On admission to the general hospital ward from the LTAC, the patient reported a 2-day history of progressive right wrist and hand pain and tenderness and right hand swelling. He complained of fatigue, shortness of breath, generalized weakness, and easy bruising and bleeding. He had no prior surgical history, no known drug allergies, and denied tobacco, alcohol, or drug use. His home medications included aspirin, atorvastatin, carvedilol, lisinopril, and as needed sublingual nitroglycerin. Transfer medications included meropenem, famotidine, furosemide, and enoxaparin 90 mg (1 mg/kg bodyweight) every 12 hours subcutaneously. It was not clear from the transfer records whether the indication for therapeutic anticoagulation with enoxaparin was the hypercoagulable state associated with COVID-19 or a suspected right arm arterial or venous thrombosis.

### 2.1. Physical Exam

His admission blood pressure was 158/70, heart rate 104, temperature 96.7°F (35.9°C), respiratory rate 30/minute, oxygen saturation 88% on 10 liters nasal cannula, weight 88 kilograms, and height 178 cm. He was alert, fully oriented, and tachypneic using accessory respiratory muscles with increased work of breathing. His exam was significant for diffuse bilateral inspiratory crackles without wheezing on lung auscultation and diffuse anasarca in all four extremities. His right wrist and hand were mildly tender and swollen with some bruising noted on the inside of the right wrist. The right radial and ulnar pulses were palpable. He had normal sensation and motor strength in the hand and wrist. The fingers of the right hand were cool to touch.

### 2.2. Laboratory and Imaging Findings

**Figure 1:**
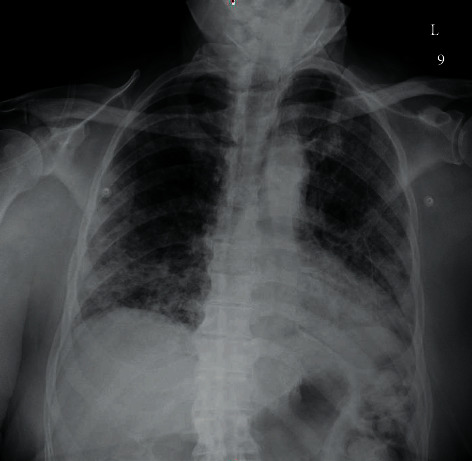
Chest X-ray anteroposterior on admission: moderately extensive bilateral patchy airspace disease.

Relevant admission labs are given in Table 1. Electrolytes and renal function were normal. The admission chest X-ray is shown in Figure 1.

### 2.3. Hospital Course and Treatment

A heparin infusion instead of therapeutic enoxaparin was instituted for suspected right upper extremity arterial thrombosis, antibiotics for suspected secondary pneumonia were broadened, and intravenous steroids for persistent severe ARDS from COVID-19 pneumonitis were restarted. He was transferred to the intensive care unit due to his profound hypoxemia and required 100% oxygen administered by both high-flow nasal cannula and nonrebreather mask to maintain oxygen saturations above 92%.

The initial differential diagnosis for the patient's right forearm and hand pain and swelling without sensorimotor symptoms included venous or arterial thrombosis; both the sending and admitting physicians had suspected arterial ischemia as the cause of his hand pain and initiated treatment with full anticoagulation, which would have been appropriate treatment for both venous and arterial thrombosis. The physical exam was not consistent with radial or ulnar arterial thrombosis, with palpable radial and ulnar pulses, though digital small artery ischemia, as can be seen with vasopressor treatment, septic shock, disseminated intravascular coagulation, or heparin-induced thrombocytopenia, remained a possibility. Arterial duplex studies demonstrated patent arterial vasculature of the right arm and digits. The vascular surgery team evaluated the patient, did not think the patient had right hand ischemia, and recommended a compression dressing to the swollen wrist. Venous thrombosis remained a concern, and venous doppler studies were pending.

20 hours after admission, the patient reported new onset numbness and tingling in his right thumb, index, and middle fingers. On exam, he had significant tenderness to palpation in the distal volar and dorsal forearm just proximal to the wrist, and the right forearm and hand were significantly more swollen than only hours earlier, with development of a significant hematoma in the right forearm; the radial pulse was no longer palpable. He had newly diminished sensation over the palmar aspect of the D1–3 fingers in a median nerve distribution, and he was unable to move his right first three digits. These findings raised concern for an acute compressive median nerve neuropathy and radial artery occlusion due to compartment syndrome of the right forearm caused by a rapidly expanding hematoma. The ICU team discontinued the heparin drip, applied external pressure to the right radial artery to prevent further bleeding, and consulted plastic surgery for fasciotomy. Compartment pressures were not measured, since the diagnosis was established clinically. The PTT was 40 seconds off heparin (normal <36 seconds); therefore, heparin reversal with protamine was not required.

The patient underwent emergent fasciotomy of the right forearm and hand and right carpal tunnel release. Intraoperatively, the volar superficial compartment had dense hematoma inside the fascial sleeves, especially in the proximal forearm, and the subcutaneous tissue had broad areas of old blood. The hemorrhage in the tissues crossed the carpal tunnel. The median nerve was encased in blood along with the volar forearm due to hematoma. Most of the muscle appeared more hemorrhagic than ischemic. The hemorrhage extended through the superficial and deep flexor musculature in the forearm. The muscles on the dorsal forearm and the extensor muscle compartment were healthy without hematoma. After fasciotomy of the dorsum of the hand, the dorsal interosseous muscles were noted to be engorged with hemorrhagic muscle. The palm and thenar area were soft. The fasciotomy extended to the entire volar forearm and half of the dorsal forearm and across the volar wrist and on the dorsum of the hand.

The patient required two more surgeries, with exploration and debridement four days later and wound exploration and closure 11 days later. His hospital course was complicated by acute blood loss anemia due to ongoing venous bleeding from the surgical site, with a hemoglobin drop to 6.8 g/dL from 12 g/dL in the first 24 hours after surgery requiring blood transfusion. The patient had persistent respiratory failure requiring mechanical ventilation for 5 days postoperatively and septic shock requiring vasopressors and empiric antibiotics for suspected hospital-acquired pneumonia. A pulmonary embolism was ruled out by CT. The venous doppler studies revealed a superficial right cephalic vein thrombosis. After aggressive intravenous diuresis for volume overload, he was extubated to BIPAP and weaned to 4 liters nasal cannula on discharge. The patient's right hand and finger weakness improved, but did not completely resolve, and he continued to require intensive occupational therapy. He was discharged to a rehabilitation facility after a combined 41-day hospital stay in 3 hospitals since his initial COVID-19 diagnosis.

## 3. Discussion

This patient developed right forearm compartment syndrome with sensorimotor function loss due to an expanding hematoma in the setting of therapeutic anticoagulation with low-molecular-weight heparin and a heparin infusion, after repeat radial artery punctures for ABG sampling had led to radial artery injury. Records from the outside hospital did not indicate how long the patient had received therapeutic enoxaparin before transfer and whether this treatment was done for suspected thrombosis or COVID-19 hypercoagulable state which many clinicians at the time treated with either therapeutic or intermediate dose anticoagulation. A small right wrist hematoma was present on admission, and the patient's hand pain was either caused by hematoma or possibly an early sign of compartment syndrome. The hematoma then expanded in the setting of ongoing empiric anticoagulation for suspected thrombosis, even though no further arterial punctures were performed. Once loss of sensation and motor function in a median nerve distribution occurred, the presence of compartment syndrome was quickly recognized, and a timely surgical intervention saved the patient's limb, with significant improvement of right hand motor and sensory function at the time of discharge.

**Figure 2:**
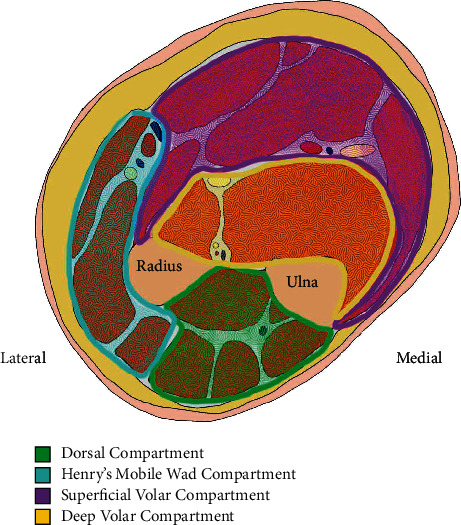
Compartments of the forearm (color). Schematic drawing by Orlando Garner, MD.

The diagnosis of compartment syndrome can be difficult to establish in its early stages, but the cardinal symptoms can be easily remembered as the 5 P's: pain, pallor, pulselessness, paresthesia, and paralysis. Compartment syndrome is treated surgically by fasciotomies of all compartments, which releases the increased pressure that causes tissue and nerve damage. The forearm consists of the following three compartments: dorsal, volar (further divided into a deep and superficial volar compartment), and “mobile wad” (Figure 2). The muscles of the dorsal compartment are innervated by the posterior interosseous nerve; the posterior interosseous artery and interosseous perforators originating from the anterior interosseous artery provide the blood supply to the dorsal compartment. The volar compartment comprised of the muscles that flex, pronate, and supinate both the wrist, hand, and fingers. The median and ulnar nerves innervate the muscles in the volar compartment, and the radial, ulnar, and anterior interosseous arteries provide its blood flow [3].

Table 2 provides cases of compartment syndrome secondary to arterial puncture for ABG sampling or arterial line insertion that have been reported in the literature. Shared characteristics include the presence of anticoagulation or a bleeding diathesis, multiple arterial puncture attempts, and the need for fasciotomy for treatment. Outcomes were mixed.

Forearm compartment syndrome has also been rarely described in the interventional cardiology literature. Cardiologists frequently use the radial artery as the access site for cardiac catheterization, since transradial catheterization has been associated with fewer major access site-related complications compared to a transfemoral approach. Other complications after transradial cardiac angiography include asymptomatic radial artery occlusion with preserved ulnar perfusion in 2–18%, radial artery spasm in 5–10%, and radial artery perforation in 0.1–1% of procedures. Pseudoaneurysms, arteriovenous fistulas, and hand ischemia are extremely rare [14]. In one case series of 520 patients undergoing transradial percutaneous coronary intervention, about 10% developed forearm hematomas; independent predictors of hematoma formation were age, body mass index, multiple puncture attempts, intensive antiplatelet therapy, and multiple catheter exchanges [15]. In another large case series from one hospital, the incidence of compartment syndrome was 0.004% or 2 out of 51,296 transradial cardiac catheterizations [16].

In summary, compartment syndrome is a rare complication of radial arterial puncture for ABG sampling, arterial line placement, or transradial cardiac catheterization. Forearm compartment syndrome can develop due to procedure-related laceration of the artery, especially if multiple attempts at arterial cannulation are performed. Risk factors for hematoma formation include insufficient external compression after arterial puncture, anticoagulation, or a bleeding diathesis, such as those associated with end-stage renal disease and end-stage liver disease. The forearm compartments are small and cannot hold a large amount of blood; therefore, nerve compression and damage can occur quickly and lead to permanent loss of function of the hand. Other potential complications associated with arterial puncture include pseudoaneurysm or arteriovenous (AV) fistula development, which often require intervention with thrombin injection or surgical excision in the case of a pseudoaneurysm or surgical patch repair in the setting of an iatrogenic AV fistula. An expanding hematoma originating from a bleeding femoral or brachial/axillary artery can lead to hemorrhagic shock, since the thigh and upper arm can hold significant amounts of blood products, in contrast to the forearm.

These complications may be avoided by providing adequate compression after an arterial puncture, by placing arterial lines when frequent ABG sampling is anticipated, by performing arterial procedures before the initiation of anticoagulation when possible, by minimizing the number of arterial puncture attempts, and by having experienced providers perform these procedures or using ultrasound guidance.

ABG sampling can provide essential information in critically ill patients with hypoxemia or hypercapnia, but it must be used judiciously; overtesting can lead to patient harm through procedure-related complications, false-positive results leading to more invasive and costly testing and iatrogenic anemia from frequent blood draws [17]. Less is often more in the ICU [18].

## 4. Conclusion and Take-Home Messages

- Even apparently routine and harmless procedures, such as obtaining an arterial blood gas, can cause serious complications. Physicians need to weigh the risks and benefits of any tests and procedures. Less is often more.
- Compartment syndrome is a devastating complication of arterial puncture and should be considered in patients presenting with extremity pain and loss of sensorimotor function after an arterial puncture
- The 5 P's of compartment syndrome are pain, pulselessness, pallor, paresthesia, and paralysis.

## Figures and Tables

**Table 1:** Labs on admission.

	Lab value	Normal range
Arterial pH	7.44	7.35–7.45
Arterial pCO_2_	41 mmHg	35–45 mmHg
Arterial pO_2_ (on high-flow nasal cannula 100% FiO_2_ 40L flow)	87 mmHg	80–90 mmHg
Hemoglobin	12 g/dL	13.7–17.5 g/dL
White blood cell count, differential	44,700/*μ*L 90% neutrophils 6% bands 1% monocytes 2% eosinophils	3,500–10,500/*μ*L
Platelet count	293,000/mm^3^	150,000–450,000/mm^3^
INR	1.4	
PTT	121 seconds (while on IV heparin)	22.5–36.0 seconds
D-Dimer	0.86 mg/L	<0.50 mg/L FEU
Total protein	6 g/dL	6.0–8.3 g/dL
Albumin	2.6 g/dL	3.5–5.0 g/dL
Total bilirubin	0.4 mg/dL	0.2–1.2 mg/dL
ALP (alkaline phosphatase)	173 U/L	40–150 U/L
AST	43 U/L	5–34 U/L
ALT	57 U/L	6–55 U/L
Lactate (arterial)	2 mmol/L	0.5–2.2 mmol/L
CK	36 U/L	29–200 U/L
CRP	0.83 mg/dL	<0.5 mg/dL
LDH	417 U/L	125–220 U/L
Blood cultures on admission	No growth	No growth
Sputum culture on admission	No growth	No growth

**Table 2:** Summary of case reports of forearm compartment syndrome after arterial puncture.

Patient characteristics	Anticoagulation or bleeding risk factors	Procedure	Diagnosis	Treatment	Outcome	Reference
29-year-old woman with pulmonary embolism	Coumadin	Unsuccessful attempt at ABG sampling from left radial artery	Left forearm compartment syndrome	Fasciotomy of the volar compartment of the left forearm; skin grafting	Full recovery of motor function; persistent small sensory deficit.	Halpern, 1978 [4]
54-year-old woman with pulmonary embolism	Thrombolysis with tenecteplase	Blood gas sampling from right radial artery	Right forearm compartment syndrome	Emergent fasciotomy; skin grafting	“Uneventful recovery”	Bisarya, 2013 [5]
30-year-old woman with end-stage renal disease due to Goodpasture's syndrome	Uremia	Blood gas sampling from left brachial artery	Left volar forearm compartment syndrome	Emergent fasciotomy; platelet transfusion for uremic platelet dysfunction	Complete recovery of sensory function 2 days after surgery	Safran, 1994 [6]
16-year-old woman after a motor vehicle accident with extensive intraabdominal and intrathoracic injuries; no right upper extremity fractures	Prolonged surgery with massive intraoperative blood loss requiring massive transfusion	Preoperative right brachial arterial line placement after several needle passes	Right forearm and hand compartment syndrome	Monitoring of compartment pressures. No surgical intervention performed.	Full recovery	Horlocker, 1995 [7]
52-year-old man with end-stage liver disease secondary to primary sclerosing	Severe intraoperative coagulopathy requiring massive transfusion during orthotopic liver transplantation	Right radial arterial line placement after 2 unsuccessful attempts	Right anterior forearm compartment syndrome	Fasciotomy	Full recovery	Lipton, 2018 [8]
Patient in 60s admitted with acute pulmonary embolism	Full dose anticoagulation with therapeutic low-molecular-weight heparin and then therapeutic heparin infusion	Multiple attempts at ABG sampling from the radial artery	Right forearm compartment syndrome	Emergent fasciotomy; extensive debridement of necrotic muscle; required a total of 6 surgeries and skin grafting	Poor outcome with poor wrist and finger flexion; impaired sensation in the median and ulnar nerve distributions in the hand	Elmorsy, 2017 [9]
ICU patient admitted with pulmonary embolism, age not reported	Full anticoagulation and thrombolytics	Multiple attempts to obtain blood gas sample from right radial artery	Right forearm compartment syndrome	Emergent fasciotomy; multiple washout and debridement surgeries	Only partial improvement of motor and sensory deficits	Elmorsy, 2017 [9]
ICU patient who developed DVT and PE during admission, age not reported	Therapeutic heparin infusion	Multiple attempts to obtain blood gas sample from right radial artery	Right forearm compartment syndrome	No surgical intervention performed due to overall poor prognosis of patient.	Death 7 days later	Elmorsy, 2017 [9]
22-year-old man admitted to ICU after severe head injury	None	Repeated arterial punctures for blood gas sampling	Large false aneurysm of the distal right radial artery with subsequent forearm compartment syndrome	Resection of the false aneurysm	Not reported	Matsagas, 2003 [10]
76-year-old man with acute coronary syndrome	Therapeutic heparin infusion after cardiac stent placement	Accidental placement of a 21-gauge intravenous cannula into the right brachial artery which was removed without immediate complications	Compartment syndrome of the flexor forearm compartment developed 2 days after accidental arterial cannulation, while on heparin infusion	Fasciotomy, repair of brachial artery tear, skin grafting	Full recovery	Shabat, 2002 [11]
4 patients (3 women, 1 man), age range 60–75	Full anticoagulation	Transradial left heart catheterization	Forearm compartment syndrome; rupture of the radial artery at the puncture site (3 patients); rupture of both brachial and radial artery (one patient)	Early surgical decompressive fasciotomy; repair of arterial ruptures	Full recovery	Lee, 2020 [12]
68-year-old man with acute coronary syndrome	Heparin infusion. Vasospasm induced by the radial arterial sheath	Cardiac catheterization via the right radial artery	Compartment syndrome caused by vasospasm	Fasciotomy; intraoperative finding of edema without any evidence of bleeding or hematoma or injury to the artery	Full recovery	Araki, 2010 [13]

## Additional Points

Even apparently harmless procedures, such as obtaining an arterial blood gas, can cause serious complications. Physicians need to weigh the risks and benefits of all tests and procedures. Compartment syndrome is a devastating complication of arterial puncture and should be considered in patients presenting with extremity pain and loss of sensorimotor function after an arterial puncture or line placement. The 5 P's of compartment syndrome are pain, pulselessness, pallor, paresthesia, and paralysis.
